# Redox Proteomic Profiling of Specifically Carbonylated Proteins in the Serum of Triple Transgenic Alzheimer’s Disease Mice

**DOI:** 10.3390/ijms17040469

**Published:** 2016-04-12

**Authors:** Liming Shen, Youjiao Chen, Aochu Yang, Cheng Chen, Liping Liao, Shuiming Li, Ming Ying, Jing Tian, Qiong Liu, Jiazuan Ni

**Affiliations:** 1 Shenzhen Key Laboratory of Marine Biotechnology and Ecology, College of Life Science, Shenzhen University, Shenzhen 518060, China; shenlim@163.com (L.S.); qyzcyj1239329@163.com (Y.C.); aochuyang@163.com (A.Y.); chenchengszu@163.com (C.C.); xiaoliping3@163.com (L.L.); yingming@szu.edu.cn (M.Y.); jtian@szu.edu.cn (J.T.); jzni@szu.edu.cn (J.N.); 2 Shenzhen Key Laboratory of Microbial Genetic Engineering, College of Life Science, Shenzhen University, Shenzhen 518060, China; shuimingli@szu.edu.cn

**Keywords:** Alzheimer’s disease, biomarker, oxidative stress, protein carbonylation, redox proteomics, serum, triple transgenic mice

## Abstract

Oxidative stress is a key event in the onset and progression of neurodegenerative diseases, including Alzheimer’s disease (AD). To investigate the role of oxidative stress in AD and to search for potential biomarkers in peripheral blood, serums were collected in this study from the 3-, 6-, and 12-month-old triple transgenic AD mice (3×Tg-AD mice) and the age- and sex-matched non-transgenic (non-Tg) littermates. The serum oxidized proteins were quantified by slot-blot analysis and enzyme-linked immunosorbent assay (ELISA) to investigate the total levels of serum protein carbonyl groups. Western blotting, in conjunction with two-dimensional gel electrophoresis (2D-Oxyblot), was employed to identify and quantify the specifically-carbonylated proteins in the serum of 3×Tg-AD mice. The results showed that the levels of serum protein carbonyls were increased in the three month old 3×Tg-AD mice compared with the non-Tg control mice, whereas no significant differences were observed in the six and 12 months old AD mice, suggesting that oxidative stress is an early event in AD progression. With the application of 2D-Oxyblot analysis, (immunoglobin) Ig gamma-2B chain C region (IGH-3), Ig lambda-2 chain C region (IGLC2), Ig kappa chain C region (IGKC), and Ig kappa chain V-V region HP R16.7 were identified as significantly oxidized proteins compared with the control. Among them IGH-3 and IGKC were validated via immunoprecipitation and Western blot analysis. Identification of oxidized proteins in the serums of 3×Tg-AD mice can not only reveal potential roles of those proteins in the pathogenesis of AD but also provide potential biomarkers of AD at the early stage.

## 1. Introduction

Alzheimer’s disease (AD) is the most important cause of dementia in the elderly and is characterized by progressive cognitive deficits that gradually lead to loss of memory and impaired activities in daily living. The pathological hallmarks of the disease are deposition of senile plaques (SP) resulting from the extracellular deposits of amyloid-β peptide (Aβ) to the intracellular neurofibrillary tangles (NFTs) caused by the aggregation of hyperphosphorylated tau protein [1]. AD is currently incurable and ultimately fatal with death typically occurring 4–6 years after clinical diagnosis [2]. Neurodegeneration in AD is estimated to start 20 to 30 years before the first clinical symptoms become apparent. Thus, therapeutic treatment might be more effective before pathological changes spread throughout the brain. It is also crucial to develop the drugs that will delay disease progression in patients with AD risk or prevent the onset of AD in normal elderly subjects [3]. Consequently, there is an increasing effort to search for the new biomarkers of AD for clinical diagnosis of the disease at its early stage.

Although AD has been discovered for over a hundred years, clinical diagnosis of the disease is still based on patient’s medical history, physical test and neurologic examination, which is confirmed by postmortem brain pathological investigation. Cerebrospinal fluid (CSF) biomarkers of AD have been developed in parallel with the markers of brain imaging techniques, such as magnetic resonance imaging (MRI) and positron emission tomography (PET) [3]. Nevertheless, CSF collection is invasive and unlikely to become a routine procedure in geriatric clinics. The biomarkers of central nervous system (CNS) are insightful but impractical for high-throughput population-based screening [4]. From a clinical perspective, the easy availability of plasma and serum samples makes their proteomic analysis the most interesting method to search for potential biomarkers that can be used in daily practice. A comprehensive and systematic characterization of plasma proteomes in healthy and diseased states could greatly facilitate the detection of biomarkers for early disease diagnosis, illness prognosis, and therapeutic monitoring [5].

In addition to changes in proteomic profiles, post-translational protein modifications of specific amino acids also occur in response to disease-associated stress. Oxidative stress has been firmly established as one of the main pathogenic events to induce and exacerbate mild cognitive impairment (MCI) and AD. It is a crucial upstream factor in the pathogenesis of the disease [6]. Previous studies have demonstrated increased levels of oxidative stress in different biomolecules, including protein oxidation, lipid peroxidation, and DNA oxidation, in the brains and blood of AD subjects compared with healthy controls [7]. Products of protein oxidation in the blood can be originated from the brain and reach the blood via the blood-brain barrier (BBB), or come from the directly oxidized proteins in the bloodstream. It has been found that BBB permeability is significantly increased in both AD and vascular dementia as compared with aging controls [8,9]. Protein carbonylated levels are a general and widely used index to determine the extent of oxidative modification of proteins both under *in vivo* and *in vitro* conditions [10]. Using a new technique of redox proteomics, Butterfield *et al.* catalogued oxidatively-damaged proteins in AD brain tissue [11]. Some specifically carbonylated proteins in AD brain were identified in different stages of the process, including the MCI and early AD stages [10,11,12,13,14,15]. This approach was also employed to elucidate possible plasma proteins in AD with specific oxidative modifications [16,17,18].

Although oxidative stress is a biochemical hallmark in AD, the associations between these markers and the pathogenic cascades involved in AD are complex and remain to be clarified. Currently the results of blood oxidative stress markers are not consistent among various studies. Many researchers found increased levels of oxidative stress markers or decreased levels of antioxidants in the blood of MCI or AD subjects, but other researchers reported non-significant results [7]. Therefore, it is important to use animal models to investigate the contradictory results related to the pathogenesis of AD. Currently, a triple transgenic mouse model of AD (3×Tg-AD) has been developed, which was derived from mixed 129/C57BL6 mice and harboring the mutated genes of APPswe, PS1M146V, and TauP301L [19,20]. Those mice exhibit both Aβ and phosphorylated tau alterations, recapitulating most of the pathological features of the AD brains [21]. In this model, Aβ dependent mitochondrial dysfunction starts at three months of age and extracellular Aβ deposits appear in the cortex at six months of age. Tau alterations are not apparent before 12 months of age [22]. In this study, to study the role that oxidative stress may play in the progression of AD and to search for the biomarkers of AD, we used male 3×Tg-AD mice and age-matched non-transgenic controls to identify carbonylated serum proteins at early and advanced AD stages (3-, 6-, and 12-month-old). Slot-blot analysis and enzyme-linked immunosorbent assay (ELISA) were used to detect the total levels of serum protein carbonyl groups and two-dimensional gel electrophoresis (2D-Oxyblots) to identify which proteins were subject to oxidation.

## 2. Results

### 2.1. Protein Carbonylated Level

**Figure 1:**
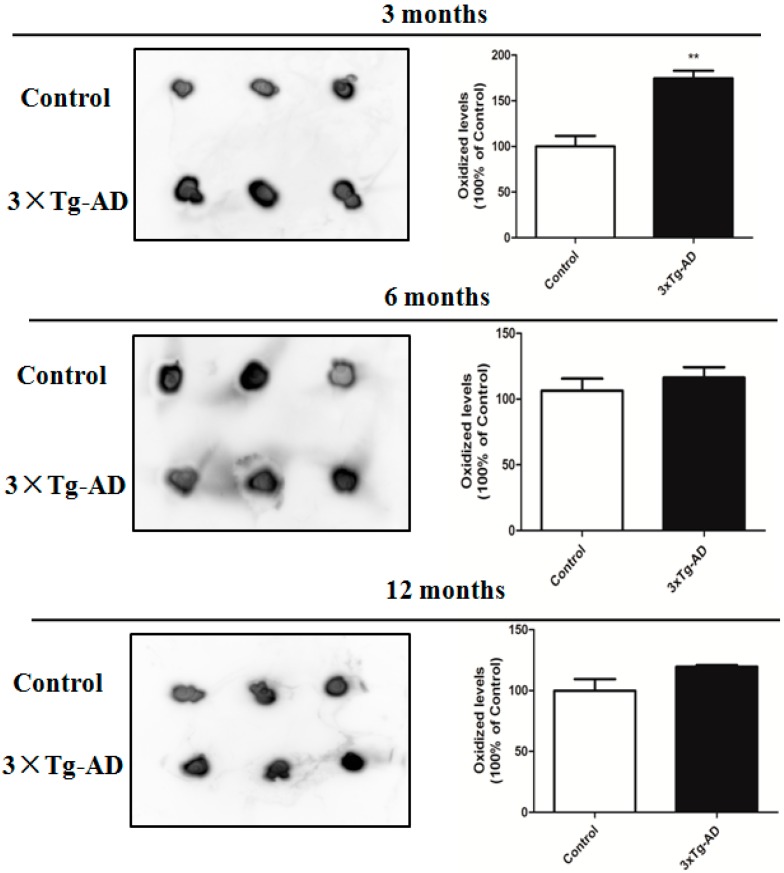
Total protein carbonylated levels in the serum of 3×Tg-AD mice compared with their controls at three, six, and 12 months of age. Slot-blot detection of protein carbonyls (**left**). The histogram shows the alteration of protein carbonylated levels (**right**). The level of protein carbonyls is normalized with the mean of the controls (*n* = 3). ** *p* < 0.05, statistically significant difference compared with the control.

Levels of protein carbonyls in the serum of 3-, 6-, and 12-month-old male 3×Tg-AD and age- and sex-matched control mice were measured by slot-blot analysis. As shown in Figure 1, increased protein carbonylated level was only detected in the serums of 3-month-old 3×Tg-AD mice compared with the non-Tg control mice (*p* < 0.05). No significant differences were observed in protein carbonylated levels between 6- or 12-month-old 3×Tg-AD mice and their age-matched non-Tg controls.

### 2.2. Detection of the Total Serum Protein Carbonylated Levels by Enzyme-Linked Immunosorbent Assay (ELISA)

**Figure 2:**
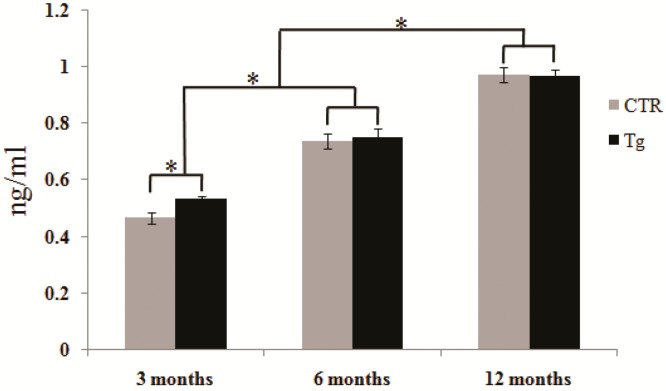
Levels of total protein carbonylated detected by enzyme-linked immunosorbent assay (ELISA). Data represent the average mean total protein carbonyl. Error bar indicates standard error of the mean (SEM) for six subjects in each group. Measured value is normalized with the mean of the control subjects. * *p* < 0.05. CTR, the control mice; Tg, the transgenic 3×Tg-AD mice.

Protein carbonylated levels were further measured by derivatization of the carbonyl groups with 2,4-dinitrophenylhydrazine (DNPH) and ELISA quantization of the DNPH group. Figure 2 show the total levels of protein carbonyl detected in the serums of 3×Tg-AD and control mice. Consistent with the results of the slot-blot analyses, the levels of serum protein carbonyls showed a significant increase in three-month-old 3×Tg-AD mice compared with the age-matched non-Tg control group (*p* < 0.05). However, at six and 12 months of ages no significant differences were observed in serum protein carbonylated levels between the 3×Tg-AD mice and their age-matched control mice. The levels of serum protein carbonyl are higher at six and 12 months of ages than that at three months of age for both 3×Tg-AD mice and non-Tg control mice.

### 2.3. Identification of Carbonylated Proteins in the Serum of 3×Tg-AD Mice

**Figure 3:**
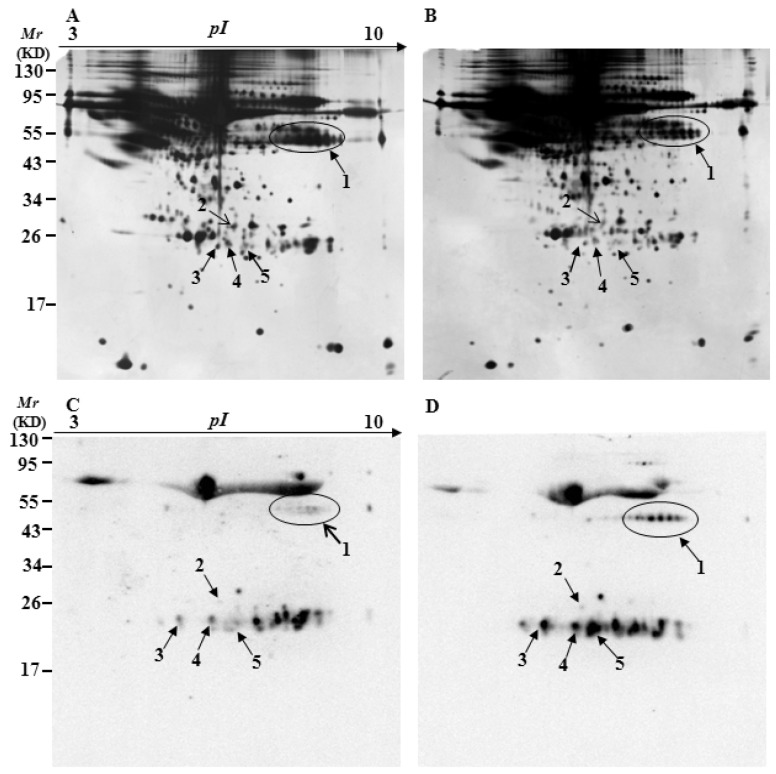
Silver-stained gels and 2D-oxyblots of serum extracts from the three-month-old controls to 3×Tg-AD mice. Representative 2D gel images of the serum proteomes of control (**A**); and 3×Tg-AD mice (**B**); (**C**,**D**) represent the oxyblots of serums from the control to 3×Tg-AD mice, respectively. Spots that showed a significant difference in specific carbonylation levels are marked with the corresponding protein identity.

**Figure 4:**
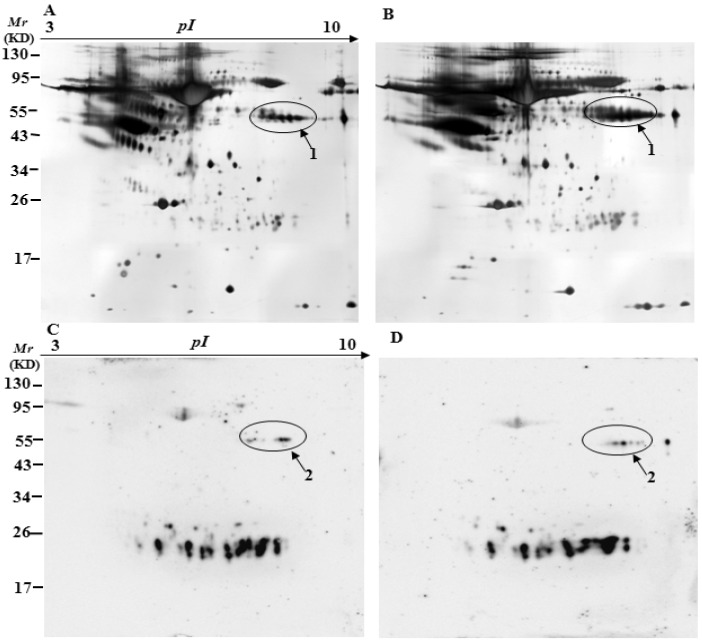
Silver-stained gels and 2D-oxyblots of the serum extracts from six-month-old controls to 3×Tg-AD mice. Representative 2D gel images of the serum proteomes of the control (**A**) and 3×Tg-AD mice (**B**); (**C**,**D**) are the oxyblots of serums from the control to 3×Tg-AD mice, respectively. Spots that showed a significant difference in specific carbonylation levels are marked with the corresponding protein identity.

**Figure 5:**
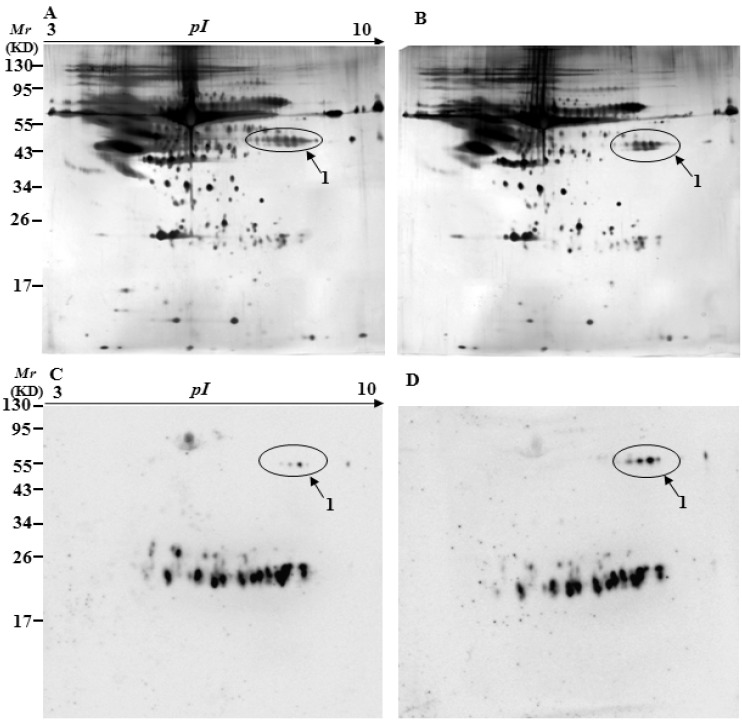
Silver-stained gels and 2D-oxyblots of the serum extracts from 12-month-old controls to 3×Tg-AD mice. Representative 2D gel images of the serum proteomes of control (**A**); and 3×Tg-AD mice (**B**); (**C**,**D**) are the oxyblots of serums from the control to 3×Tg-AD mice, respectively. Spots that showed a significant difference in specific carbonylation levels are marked with the corresponding protein identity.

To identify specifically carbonylated proteins in the serum, after separation by two-dimensional gel electrophoresis (2-DE), one gel was stained with silver for total proteins and the other was immunostained for oxidized proteins. Figure 3, Figure 4 and Figure 5 show representative 2D gels and the corresponding 2D-Oxyblots of the serums from 3-, 6-, and 12-month-old controls and 3×Tg-AD mice, respectively. The identified specifically-carbonylated proteins are listed in Table 1. Compared with the corresponding aged-matched controls, five, one, and one protein were found to have increased carbonylation in 3-, 6-, and 12-month-old AD mice, respectively. No significant differences were observed in protein expression level between the 3×Tg-AD mice and control groups. Spots 3 and 4 were identified to be the same protein (Table 1, Figure 3). The results show that Ig gamma-2B chain C region (IGH-3), Ig lambda-2 chain C region (IGLC2), Ig kappa chain C region (IGKC) and Ig kappa chain V-V region HP R16.7 are significantly oxidized in the serums of three-month-old 3×Tg-AD mice compared with that in the serums of age-matched control mice (Table 1, Figure 3). Among them, IGH-3 also shows significantly higher carbonylated levels in six, 12-month-old 3×Tg-AD mice serum, as compared with their non-Tg controls (Table 1, Figure 4 and Figure 5).

Interestingly, the results of 2D-Oxyblots showed that the pattern of oxidized proteins was similar in 3×Tg-AD mice at six and 12 months of age when compare with their age-matched controls, whereas significant differences were observed between 3×Tg-AD and control mice at three months of age (Figure 3, Figure 4 and Figure 5), which is similar to the results from slot blot analysis to ELISA assay as described above. Comparison of the results from three different detection methods shows similar pattern and trend of oxidized proteins in the serums of AD mice.

### 2.4. Validation of Redox Proteomics Results

**Figure 6:**
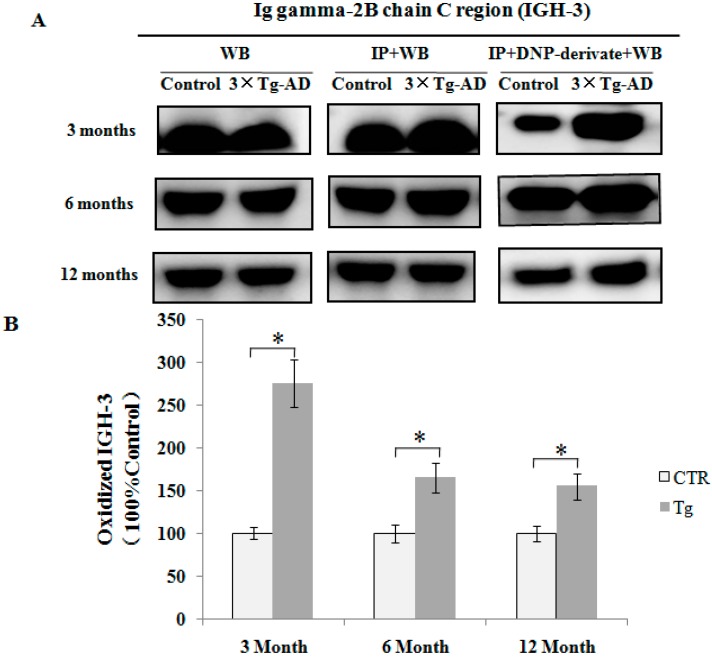
Immunoprecipitation followed by Western blot analysis was performed to confirm the carbonylation of IGH-3 proteins in serum. (**A**) The expression level of the protein was detected with anti-IGH-3 (WB). The efficiency of immunoprecipitation was checked with anti-IGH-3 (IP + WB). The protein was immunoprecipitated with its antibody and Western blot analysis with anti-DNP antibody (IP + DNP-derivate + WB); (**B**) Histograms shows the alteration of protein carbonylated levels, in which the measured value is normalized with the mean of the control subjects (*n* = 3). * *p* < 0.05 *vs.* the control.

**Figure 7:**
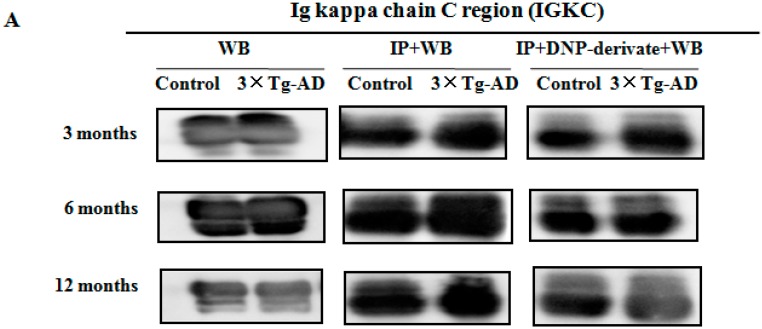

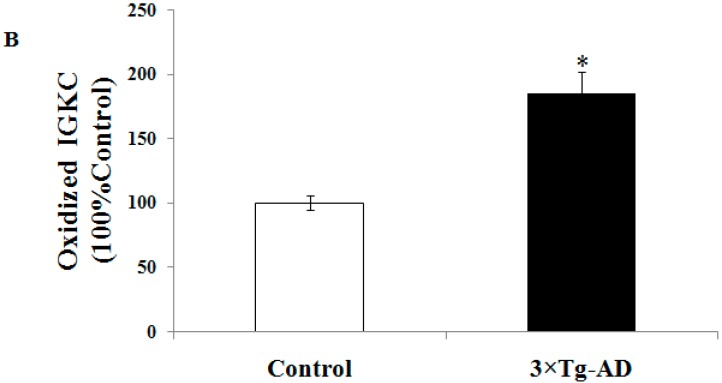
Immunoprecipitation followed by Western blot analysis was performed to confirm the carbonylation of IGKC protein in serum. (**A**) The expression level of the protein was detected with anti-IGKC antibody (WB). The efficiency of immunoprecipitation was checked with anti-IGKC antibody (IP + WB). The protein was immunoprecipitated with its antibody and Western blot analysis with anti-DNP antibody (IP + DNP-derivate + WB); (**B**) histograms show the alteration of protein-carbonylated levels, in which the measured value is normalized with the mean of the control subjects (*n* = 3). * *p* < 0.05 *vs.* the control.

In order to validate redox proteomics results, the carbonylated levels of IGH-3 and IGKC were detected using the method described previously [23]. Samples were post-derivatized with DNPH on the membrane and probed with anti-DNPH antibody to identify the oxidized proteins. As shown in Figure 6, consistent with proteomics results, the carbonylated levels of IGH-3 were significantly increased in the serum of 3×Tg-AD mice compared to controls at different months of age (*p* < 0.05). The carbonylated levels of IGKC are significantly higher in the serum of the three-month-old 3×Tg-AD mice compared with the controls (Figure 7, *p* < 0.05). No significant differences were observed in their protein expression levels between the 3×Tg-AD mice and control groups (Figure 6 and Figure 7).

## 3. Discussion

To elucidate the molecular changes that occur in blood and search for the biomarker, in this study, we quantified the levels of protein carbonyls and identified which proteins were subject to oxidation of AD pathology at different stages of the disease using the 3×Tg-AD mouse models. Our results showed that the levels of total protein carbonyls in the serums change age-dependently. Significant difference between 3×Tg-AD mice and their respective controls were observed at three months of age but not at naval and 12 months of age. The results suggest that oxidative stress occurs as early as three-month-old in the 3×Tg-AD mice, which is an early event in the development of AD and precedes the accumulation of Aβ in senile plaques and the formation of NFTs. Oligomers of Aβ have been heavily implicated in the initiation and pathogenesis of AD, while monomeric forms of Aβ have been suggested to be less harmful, and even neuroprotective [24]. 3×Tg-AD mice start Aβ oligomerization at 2–6 months old [25]. Thus, the oxidative stress observed in the three-month-old mice could possibly be initiated by oligomeric Aβ [24]. Recently, a meta-analysis [26] on nine studies [27,28,29,30,31,32,33,34,35] showed that two of them described had increased levels of carbonylation at this stage, which is in MCI with cognitive decline [29,35]. This is in agreement with our results in this study that the increased level of protein carbonyl was only observed in the early stage of AD. Very recently, we have reported that increased protein carbonyls were observed in the hippocampi of 3×Tg-AD mice early at three months of age. As a compensatory response, carbonyl reductase 1 (CBR1), an enzyme that catalyzes reduction of protein carbonyl groups, was significantly up-regulated in the hippocampi of 3×Tg-AD mice compared with the non-Tg controls [36]. Thus, our data from both hippocampus to serum support the viewpoint that oxidative stress is an early event in AD progression [22]. The possibility of using antioxidants for prevention and treatment of AD has attracted considerable attention [37], particularly the beneficial effects at early preclinical stages of the disease. A recent study showed that the antioxidant selenomethionine (Se-Met) could improve cognitive impairment, reduce the level of total tau, inhibit tau hyperphosphorylation and ameliorate both the inflammatory response and oxidative stress in 3×Tg-AD mice [38]. It is worth noting that iron dyshomeostasis and associated redox activity/oxidative stress are also the key factors contributing to AD pathogenesis. A recent study showed that the iron chelator deferiprone conferred important protection against AD in a cholesterol-fed rabbits by reducing Aβ generation, tau hyperphosphorylation and plasma iron levels [39]. However, deferiprone failed to reduce the levels of reactive oxygen species (ROS) and intracellular H_2_O_2_, suggesting that oxidative stress generated in this AD model may result from pathways independent from Aβ generation and tau phosphorylation. Therefore, the combination of antioxidant and iron-chelator to prevent ROS generation would be more powerful to protect against the deleterious effects of AD pathology, especially in pre-clinical trial.

The meta-analysis also indicated that no significant change occurred in protein oxidation levels in blood from subjects with AD [26]. However, existing studies on the level of protein carbonylation in the blood of AD showed conflicting results, including the nine studies in the meta-analysis [26]. For example, one study showed that the levels of protein carbonyl were significantly higher in the plasma of AD/MCI subjects compared with that of healthy controls [28,29,30,32,40]. Nevertheless, the other studies showed no significant difference in the levels of serum/plasma protein carbonyl between the AD subjects and controls [27,31,34,35,41]. As oxidative stress also occurs in other diseases, such as diabetes, cardiovascular disease and other neurodegenerative diseases, it is reasonable that the results of oxidative stress markers in the blood of AD patients are not consistent in various studies [7]. In our study, no significant differences were observed in protein-carbonylated levels between 3×Tg-AD mice and controls at the ages of six and 12 months, suggesting that age is an important factor related to the change of oxidative stress. It has been reported that the total protein carbonyl content of the plasma significantly increased with age in mice [42], and increased protein oxidation has also been demonstrated in normal aging people [43]. Thus, age may be another reason that leads to the conflict of results published in the literature. However, although no differences were observed in the level of total serum protein carbonyl, significantly higher carbonylated levels of specific protein IGH-3 were observed in the serums of 6- and 12-month-old 3×Tg-AD mice compared with the controls, supporting the deduction that oxidative stress within AD are specific and critical events that take place to alter the function of certain proteins, rather than to make oxidative damage in a non-specific and random manner [26]. Consequently, if only a few proteins were identified as carbonylated in the blood of AD, no significant difference could be observed in the level of total protein carbonyls between AD and control subjects. In contrast to six- and 12-month-old 3×Tg-AD mice, more proteins were identified as specifically-carbonylated in the serum of three-month-old 3×Tg-AD mice, thus significant difference was observed between the AD and control mice.

Therefore, the detection of total serum protein carbonylated levels may not be specific to AD and MCI. This makes difficult to recommend for routine clinical use. Analyses of specific protein oxidations in the serum may be more useful in searching for the biomarkers of AD. Interestingly, several carbonylated proteins have been identified in the plasma of AD patients by the use of 2D-Oxyblots, including fibrinogen gamma-chain precursor protein, alpha-1-antitrypsin precursor [17], isoforms of human transferrin, hemopexin, and alpha-1-antitrypsin [16]. Particularly, analyses of both protein expression level and oxidative modification (carbonylation) could increase specificity of the marker. For example, a recent study showed that haptoglobin, one of the most abundantly secreted glycoproteins, was found to be either increasingly down-regulated or increasingly oxidized in AD and MCI compared with controls, indicating that it may be used as a biomarker with high specificity in plasma of patients with AD [18]. In our study, IGH-3 was identified as the protein with oxidative modification in the serum of 3×Tg-AD mice at both six and 12 months of age, suggesting that the carbonylated level of this protein may be a putative marker of AD. Meanwhile, IGLC2, IGKC and Ig kappa chain V-V region HP R16.7 were found to be specifically-carbonylated in the serum of 3-month-old mice, suggesting that they may serve as potential biomarkers for the early stage of AD. Additional studies are necessary to validate these results using serum samples from MCI to AD patients together with the age-matched healthy controls. In addition, given the complexity of AD pathogenesis, it has been speculated that a panel of proteins may perform better than any single protein to indicate the stage and change of AD progression [44,45]. Thus, these modified proteins may also be considered as one type of biomarkers in blood for AD diagnosis. As all of the identified proteins in this study belong to immunoglobulin, it is reasonable to suggest that immunoglobulin in blood is prone to be oxidized in the early stages of AD and may be important in the progression of AD. Consistent with this finding, previous studies showed that immunoglobulin G (IgG) was quite vulnerable to reactive oxygen species [46] and the elevated levels of oxidized IgG were observed in the patients of rheumatoid arthritis [46,47,48], end-stage renal disease [49] and type 1 diabetes mellitus [50]. However, in these studies [46,47,48,49,50], IgG was purified from plasma by an affinity column to the oxidized IgG was detected by one-dimensional gel electrophoresis plus western blot analysis (1D-Oxyblot). In our study, 2D-Oxyblot analysis was used so that multiple proteins with specific carbonylation were detected. In addition, these immunoglobulins were detected as oxidized proteins in blood partly due to the reason that they were not depleted by the abundant proteins depletion kit, e.g., albumin and IgG depletion kit. This suggests that although removing high-abundance proteins allows improving detection of less abundant proteins, some high-abundance proteins such as immunoglobulin may be useful in discriminating AD from the controls and involved in the pathogenesis of AD. Thus, it is also worthy to perform proteomic analysis without depletion of abundant serum proteins for screening AD biomarker. Regarding the difference between our results and previous reports [16,17,18] in the identification of carbonylated plasma proteins, it is possibly caused by the collection and pretreatment of blood sample. In those reports, the plasma samples were fractionated by sequential affinity chromatography [16] and depleted using a ProteoPrep Blue albumin and IgG depletion kit [18]. Those methods are different from ours and likely lead to the difference in protein cabonylation. Moreover, oxidative modification mainly occurs in the C region of immunoglobulin. This is consistent with previous reports that the Fc fragment may be a particularly susceptible region of the IgG molecule for oxidative modification [51]. A recent report showed that eight isoforms of different immunoglobulin chains were identified as oxidized proteins in human plasma, all of which belong to the C region of immunoglobulin chains and three proteins of them are similar to our finding, including Ig gamma-2 chain C region (IGHG2), Ig kappa chain C region (KAC), and Ig lambda chain C region (LAC) [52].

The oxidative modification of proteins has been demonstrated to play a major role in a number of pathological processes. Oxidation changes the chemical properties of amino acid side chains, leading to modification of the protein structure and folding. In many cases, these changes result in protein impairment, but some of them may instead cause protein activation [53]. However, only a few reports were about the relation between oxidized immunoglobulins and AD pathology, which were linked to amyloid deposits. For example, immunoglobulin λ light chains (λ chains) have been reported to be involved in primary immunoglobulin-related (amyloid light chain or AL) amyloidosis and were identified as oxidized proteins in the CSF of AD [54]. Interestingly, in this study the oxidation of Ig lambda-2 chain C region (IGLC2) was observed in the serums of 3×Tg-AD mice. In addition, immunoglobulin may also be deposited in tissue. For example, IgG and IgM have been shown to deposit in the epithelial basement membrane of choroid plexus in AD brain [55]. Thus, oxidized immunoglobulins may play important roles in the development of AD and needs further investigation.

Intravenous immunoglobulin (IVIG) products prepared from the blood of healthy donors have been observed to contain antibodies to Aβ, suggesting that it might be helpful in the treatment or prevention of AD [56]. IVIG can inhibit complement activation, neutralize inflammatory cytokines, and modulate chemokine expression and regulatory T cell subsets. IVIG has been used to treat a range of autoimmune, infectious and idiopathic disorders, but it is not approved for Alzheimer’s disease and negative results were reported in a phase III IVIG study [57]. Those disappointing results have reduced people’s enthusiasm for developing IVIG as a possible treatment for AD; however, it is still too early to draw final conclusions [57]. Several suggestions have been proposed for IVIG, such as earlier IVIG treatment, increasing concentration and anti-inflammatory activity, generation of IVIG products with recombinant technology, using the IVIG polyclonal antibody approach, *etc*. All of those were in an effort to deplete aggregated Aβ species [57]. Here, our results suggested that oxidatively-modified immunoglobulins and the resulting alteration in biological properties may be another reason for the failures of IVIG trials. This can be at least partly supported by the fact that the main anti-inflammatory effects of IVIG are due to its IgG Fc fragments [58,59], and *in vitro* oxidation of IgG impairs its ability to bind to macrophage Fc receptors [51]. Based on previous reports and our results, we speculate that this carbonylated modification of IVIG decreases the anti-inflammatory property of Fc fragment, leading to the requirement for high doses of IVIG to generate anti-inflammatory effects [60]. Hence, the combination of antioxidant therapy with IVIG treatment may be beneficial in the intervention of AD at its initial stage.

## 4. Experimental Section

### 4.1. Reagents

The materials were purchased from the following companies: 2,4-dinitrophenylhydrazine (DNPH) and anti-DNP (dinitrophenylhydrazone) monoclonal antibody from Sigma-Aldrich Co. (St. Louis, MO, USA); anti-IGH-3 and anti-IGKC antibody from AbcamInc (Cambridge, MA, USA) and Bioss Biotechnology Co., Ltd. (Beijing, China), respectively; anti-mouse and anti-rabbit secondary antibodies of horseradish peroxidase (HRP)-linked IgG from AbmartInc (Shanghai, China). All other reagents, except otherwise noted, were obtained from Sigma-Aldrich.

### 4.2. Sample Collection and Preparation

The experiments were performed using 3×Tg-AD (strain: B6; 129-Psen1^tm1Mpm^Tg (AβPPSwe, tauP301L) 1Lfa/Mmjax) and non-Tg mice (strain: B6129SF2/J). They were purchased from Jackson Laboratories (Bar Harbor, ME, USA). Animal treatment and housing were performed in accordance with the Guide for Care and Use of Laboratory Animals released by the National Institutes of Health [61]. Six animals for each age group (3-, 6-, and 12-months-old, male) of both 3×Tg-AD mice and controls were employed in this study. After anaesthetized with sodium pentobarbital (50 mg/kg) intraperitoneally, blood samples were collected in glass tubes without additive and allowed to clot at room temperature for 30 min. Serum was separated by centrifugation at 3000× *g* for 10 min. Aliquots of serum were collected and stored at −80 °C until use. Although abundant serum proteins are generally considered poor markers of diseases, carbonyl-modification of these proteins may be of interest. Therefore, the most abundant serum proteins were not depleted prior to analysis. Slot-blot analysis and ELISA assay were performed on three and six aqueous serum samples obtained from 3×Tg-AD to control mice, respectively. For detection of the carbonyl proteins by 2D-Oxyblots, serum samples were obtained and pooled from six 3×Tg-AD to non-Tg mice, respectively. The experiments were performed in triplicate.

### 4.3. Slot Blot Analysis for the Detection of Protein Carbonyls

Protein carbonyls were detected by slot blot analysis with anti-DNP antibody as previously described [14] with minor modifications. Serum proteins (20 µg) were treated with an equal volume of 12% sodium dodecyl sulfate (SDS) and derivatized with 10 µL of 10 mM 2,4-DNPH for 20 min. The reaction was stopped by addition of neutralizing reagent (7.5 µL of 2 M Tris/30% glycerol buffers, pH 8.0). This neutralized sample solution (250 ng protein) was loaded in each well on a polyvinylidenedifluoride (PVDF) membrane. The membrane was blocked with 5% bovine serum albumin (BSA) in phosphate-buffered saline (PBS), and then hybridized overnight at 4 °C with a 1:1000 dilution of anti-DNP antibody in PBS containing 0.2% (*v*/*v*) Tween 20 (PBST) and 5% BSA. The membrane was washed three times in PBST at intervals of 5 min each, and then incubated with a HRP-conjugated goat anti-mouse secondary antibody diluted in PBST containing 5% BSA (1:5000 dilution) for 2 h. The membrane was washed three times in PBST for 5 min each time. The DNP-derivatized (oxidized) proteins were visualized with an ECL kit (Pierce ECL detection kit, Thermo Fisher Scientific Inc., Rockford, IL, USA). Following exposure to the chemiluminescent chemicals, the signal was detected by a Kodak Image Station 4000MM imaging system (Carestream Health, Inc., Rochester, NY, USA). Images were analyzed using the Quantity One software (Bio-Rad, Hercules, CA, USA).

### 4.4. Measurement of the Total Serum Protein Carbonylated Levels Using ELISA Assay

Protein carbonyl measurements were performed by ELISA according to a previous study with minor modifications [62]. Briefly, oxidized BSA containing additional carbonyls was prepared for use as reference by reacting BSA (50 mg/mL in phosphate-buffered saline (PBS)) with hypochlorous acid (5 mM of final concentration) for 1 h at 37 °C, followed by overnight dialysis against PBS at 4 °C. For fully reduced BSA, a 10 mg/mL natural BSA solution in PBS was used to react with 1 mg/mL sodium borohydride. Serum samples were adjusted to a protein concentration of 5 µg/mL with PBS, 200 µL of the sample or blank (PBS without protein) was added in triplicate into the wells of plate and incubated overnight at 4 °C. Those samples were derivatized with DNPH (0.05 mM, pH 6.2) and then washed five times with 300 µL PBS:ethanol (1:1, *v*/*v*) solution and one time with 300 µL PBS. After incubation with blocking solution (5% skimmed milk in PBS) for 1.5 h at room temperature and washed with 300 µL PBS (0.1% Tween 20), protein carbonyls were detected using mouse anti-DNP antibody (1:1500 dilution of antibody and incubation for 1 h at 37 °C) and HRP-conjugated secondary antibody (1:4000 dilution and incubation for 1 h at 37 °C). Immunoreactivity was determined by measuring the conversion of 3,3′,5,5′-tetramethylbenzidine (TMB, Sigma-Aldrich) at 450 nm after termination of the reaction with 2 M HCl.

### 4.5. 2D-Oxyblot for Detection of the Specifically-Carbonylated Protein in the Serum

Protein carbonyls were analyzed by 2D-Oxyblot using the in-strip derivatization technique of Conrad *et al.* [36,63]. Whole protein lysate (150 μg) were mixed with rehydration solution (8 M urea, 2% CHAPS, 0.2% DTT, 2% (*v*/*v*) IPG buffer, 0.002% bromophenol blue) to a final volume of 240 µL. Precast 13-cm immobilized pH gradient (IPG) strips (non-linear (NL), pH = 3–10) (GE Healthcare, Uppsala, Sweden) were rehydrated for 12 h at 30 V. IEF (isoelectric focusing) were performed with the following voltage program: 100 V/2 h, 200 V/1 h, 500 V/1 h, linear ramp to 1000 V over 1 h, 8000 V over 3 h, then 8000 V constant for a total focusing time of 55,000 Vh [64]. After rehydration IEF, IPG strips were incubated in 2 M HCl with 10 mM DNPH for 10 min at room temperature. The strips were washed with 2 M Tris/30% (*v*/*v*) glycerol for 15 min, then reduced and alkylated [64]. The second dimensional SDS-PAGE was performed on homemade 12% polyacrylamide gels using SE 600 Ruby system (GE Healthcare) [64]. Each sample was electrophorized in duplicates. After electrophoresis, proteins in one gel were silver stained using a modified silver staining method [65], and proteins in another gel were transferred onto PVDF membrane using a TE42 Protein Transfer Tank (GE Healthcare) at 45 mA per gel for 2 h. The membranes were subsequently blocked, washed, and incubated overnight at 4 °C for immunoblotting with anti-DNP antibody (1:1000 dilution) in PBS containing 5.0% non-fat dry milk. The blots were subsequently washed with PBS and 0.2% (*v*/*v*) Tween-20 (PBST) and incubated with the goat anti-mouse IgG/HRP conjugate (1:5000 dilution) for 2 h at room temperature. After three washes with PBST, the immune complexes were visualized by enhanced chemiluminescenceas described above. Silver-stained gels were imaged using the proXPRESS 2D imaging system (PerkinElmer, Waltham, MA, USA). ImageMaster 2D Platinum software (version 5.0, GE Healthcare) was used to compare protein oxidation between the control and 3×Tg-AD samples. Relative spot volume (%V) was used for analysis, where V is the integration of optical density (OD) values over the spot area and %V is the ratio of V_single spot_/V_total spots_. The intensity of carbonylated spots on the 2D-Oxyblots was normalized *vs.* their corresponding spots visualized on the silver stained gels. The spots showing a significant difference (*p* ≤ 0.05) in carbonylation levels were chosen for protein identification.

### 4.6. Protein Identification by Mass Spectrometry (MS)

For protein identification, the spots of interest were excised manually from the silver-stained gels and tryptic in-gel digestion was performed [65]. Briefly, gel chips were destained in a 1:1 solution of 30 mM potassium ferricyanide and 100 mM sodium thiosulfate, and then equilibrated in 50 mM ammonium bicarbonate (NH_4_HCO_3_) at pH 8.0. After hydrating with acetonitrile (ACN) and drying in a Speed Vac, the gels were rehydrated in a minimal volume of trypsin solution (10 µg/mL in 25 mM NH_4_HCO_3_) and incubated at 37 °C overnight. The supernatant was directly used and the mass spectroscopy analysis was performed on a 5800 MALDI TOF/TOF mass spectrometer (AB SCIEX, Framingham, MA, USA) [66]. Peptide mass spectra were acquired in positive ion reflection mode, and 800–4000 *m*/*z* mass range with 1000 laser shots was used. Combined MS and MS/MS spectra were searched against the SwissProt database (release 2013_12) using Mascot (Matrix Science, London, UK).

### 4.7. Immunoprecipitation and Post-Western Blot Derivatization

To confirm the redox proteomics results, IGH-3 and IGKC were immunoprecipitated using IGH-3 and IGKC antibodies respectively, and then probed for protein carbonylated levels [23]. In short, protein samples (300 µg) were incubated overnight at 4 °C with the specified antibodies. Protein A and G plus-agarose beads (Santa Cruz Biotechnology Inc., Santa Cruz, CA, USA) were added and the mixture was incubated for 3 h, followed by triple washes with lysis buffer. The beads were re-suspended in SDS loading buffer and boiled for 5 min. After centrifugation, the supernatant was collected, separated by 12% SDS-PAGE gel electrophoresis, and transferred to PVDF membrane [67]. The membranes were equilibrated in solution A (20% (*v*/*v*) methanol: 80% (*v*/*v*) PBST) for 5 min, followed by incubation in 2 M HCl for 5 min. The proteins on blots were then derivatized in solution B (0.5 mM DNPH in 2 M HCl) for exactly 5 min as described by Conrad *et al.* [68]. The membranes were washed three times in 2 M HCl for 5 min each and then five times with 50% methanol and two times with PBST. The immune complexes were revealed by enhanced chemiluminescence as described above.

### 4.8. Statistical Analysis

All data are presented as mean ± SD. Differences between control and 3×Tg-AD mice were determined using Student’s two-tailed independent *t*-test. Comparisons between multiple groups were performed using the one-way analysis of variance (ANOVA) followed by LSD test. *p* ≤ 0.05 was considered significantly different.

## 5. Conclusions

In the present study, we evaluated the levels of total protein carbonyls and identified the oxidative modification proteins in the serums of 3×Tg-AD mice. AD mouse models offer great advantages towards understanding the mechanism of this disease. Our results suggested that oxidative stress is an early event in the development of AD, and analysis of specific serum protein oxidation may be more plausible for the search of AD biomarkers. By redox proteomics, four specifically-carbonylated proteins were identified in the serum of AD mice. These proteins may be potential biomarkers of AD and may contribute to the pathophysiology of AD. These results suggest that antioxidant therapy may be beneficial in the treatment of AD at the early stage. This study provides valuable information not only for understanding the pathogenesis and progression of AD, but also for discovering potential biomarkers of AD.

## Figures and Tables

**Table 1:** Proteomic identification of oxidatively-modified serum proteins at three months, six months, and 12 months of age from 3×Tg-AD mice compared with the aged-matched controls.

Age of Mice	Spot Number	Protein Identified	SwissProt Accession	*M*w (kDa)/*pI*	Protein Score	Peptides Matched ^a^	Coverage Rate (%) ^b^	Specific Oxidation (Fold) ^c^
3 months	1	Ig gamma-2B chain C region (IGH-3)	P01867	50/6.1	122	1 (1)	3	3.42
	2	Ig lambda-2 chain C region (IGLC2)	P01844	11.4/5.86	216	3 (3)	47	3.69
	3	Ig kappa chain V-V region HP R16.7	P01644	12/7.97	255	3 (3)	43	4.98
	4	Ig kappa chain V-V region HP R16.7 ^d^	P01644	12/7.97	365	4 (4)	43	2.1
	5	Ig kappa chain C region (IGKC)	P01837	12/5.23	132	2 (2)	28	4.22
6 months	1	Ig gamma-2B chain C region (IGH-3)	P01867	50/6.1	122	1 (1)	3	3.13
12 months	1	Ig gamma-2B chain C region (IGH-3)	P01867	50/6.1	122	1 (1)	3	5.3

^a^ Peptides matched by mass fingerprinting; ^b^ protein sequence coverage; ^c^ match between 3×Tg-AD mice and the control (3×Tg-AD *vs.* CTR), *p* < 0.05; ^d^ the Spots 3 and 4 were identified as the same protein.
